# Strong Photoluminescence Enhancement of Silicon Oxycarbide through Defect Engineering

**DOI:** 10.3390/ma10040446

**Published:** 2017-04-23

**Authors:** Brian Ford, Natasha Tabassum, Vasileios Nikas, Spyros Gallis

**Affiliations:** Colleges of Nanoscale Science and Engineering, SUNY Polytechnic Institute, Albany, NY 12203, USA; brford@sunypoly.edu (B.F.); NTabassum@sunypoly.edu (N.T.); vnikas@sunypoly.edu (V.N.)

**Keywords:** luminescent materials, nanowires, luminescence, spectroscopy, characterization, advanced techniques

## Abstract

The following study focuses on the photoluminescence (PL) enhancement of chemically synthesized silicon oxycarbide (SiC_x_O_y_) thin films and nanowires through defect engineering via post-deposition passivation treatments. SiC_x_O_y_ materials were deposited via thermal chemical vapor deposition (TCVD), and exhibit strong white light emission at room-temperature. Post-deposition passivation treatments were carried out using oxygen, nitrogen, and forming gas (FG, 5% H_2_, 95% N_2_) ambients, modifying the observed white light emission. The observed white luminescence was found to be inversely related to the carbonyl (C=O) bond density present in the films. The peak-to-peak PL was enhanced ~18 and ~17 times for, respectively, the two SiC_x_O_y_ matrices, oxygen-rich and carbon-rich SiC_x_O_y_, via post-deposition passivations. Through a combinational and systematic Fourier transform infrared spectroscopy (FTIR) and PL study, it was revealed that proper tailoring of the passivations reduces the carbonyl bond density by a factor of ~2.2, corresponding to a PL enhancement of ~50 times. Furthermore, the temperature-dependent and temperature-dependent time resolved PL (TDPL and TD-TRPL) behaviors of the nitrogen and forming gas passivated SiC_x_O_y_ thin films were investigated to acquire further insight into the ramifications of the passivation on the carbonyl/dangling bond density and PL yield.

## 1. Introduction

Silicon (Si)-based luminescent materials and nanostructures have attracted appreciable attention in recent years due to their potential utilization in a vast amount of technological applications [1,2]. While crystalline Si is the most important semiconductor material for the electronics industry, its optical properties are relatively poor due to its indirect band gap, preventing efficient emission and absorption of light [1]. Silicon oxycarbide (SiC_x_O_y_) materials have been extensively studied, owing to their compatibility with technologies currently used in the Si semiconductor industry, as low-k dielectrics, passivation layers, and etch-stop layers [3,4].

Furthermore, there is ongoing research in employing SiC_x_O_y_ materials and nanostructures in a diverse range of applications, displaying favorable properties for use in white light emitting materials [5,6], hydrogen storage materials [7], gas sensors [8], and even in biomedical devices [9]. Towards this, SiC_x_O_y_ nanowires (NWs) have been recently shown to exhibit strong room-temperature visible light emission [10,11]. Additionally, SiC_x_O_y_ has demonstrated to be a suitable host material for optically active rare-earth ions [12,13]. Owing to its chemical inertness and biocompatibility, SiC_x_O_y_ thin films and NWs may thus be considered as potential candidates for employment in integrated photonic and biosensing applications.

However, potential obstacles for practical application of ultra-thin films and nanostructured materials for light emission applications include their sensitivity to surface chemistry, and their low emission yield caused by charge-carrier dissociation associated with surface defects [14]. Furthermore, unpassivated surfaces of nanostructures and ultra-thin films have exhibited low chemical ambient stability due to an increase in surface defects [15]. Surface passivation/functionalization treatments have shown to significantly enhance the luminescence efficiency [16].

To this end, prior work by researchers has been dedicated to the development of a chemical vapor deposition (CVD) synthesis strategy for the growth of SiC_x_O_y_ thin films and NWs for light emission applications [10,17]. It has been demonstrated that the as-deposited SiC_x_O_y_ thin films follow closely to the pure silicon oxycarbide stoichiometry [SiC_x_O_2(1-x)_, (0 < x < 1)]. The compositional evolution of the SiC_x_O_y_ materials spans from SiC to SiO_2_, for respectively, x = 1 and x = 0. The resulting materials exhibit strong white light emission, originating from the recombination of photo-generated carriers between the energy bands and/or at their tail states, associated with the Si–O–C/Si–C bonding configurations [11,17].

This report describes the effects of post-deposition passivation treatments in oxygen (O_2_), nitrogen (N_2_), and forming gas (FG, 5% H_2_ and 95% N_2_) ambients on the structural and photoluminescence (PL) characteristics of two compositions of amorphous SiC_x_O_y_ materials. The white light emission from both the as-deposited and passivated materials was strong enough to be seen by the naked eye in bright room conditions, and the integrated PL intensity was found to be related to the carbonyl (C=O) bonding present in the films.

## 2. Results and Discussion

**Figure 1:**
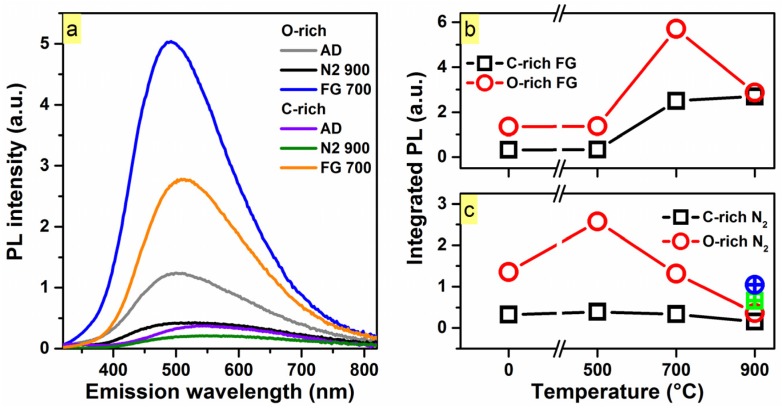
(**a**) Room-temperature steady-state photoluminescence (PL) spectra for the as-deposited, N_2_ 900°C, and forming gas (FG) 700 °C O-rich SiC_0.3_O_1.6_ and C-rich SiC_0.5_O_1.3_ matrices under 300 nm (4.13 eV) excitation. More than one order of magnitude (~17.8 and ~16.6 times for the O-rich and C-rich respectively) difference in PL (peak-to-peak) was observed between passivated samples; (**b**) The integrated PL (*I_PL_*) as a function of FG-passivation temperature for both matrices. The maximum increase in *I_PL_* was found to be at 700 °C for the O-rich matrix while the FG-passivation for the C-rich matrix yielded a continuous luminescence increase at 900 °C; (**c**) The *I_PL_* as a function of N_2_-passivation temperature for both matrices. For both compositions of SiC_x_O_y_, it was found that higher temperature N_2_-passivations yielded a decrease in observed *I_PL_*. After passivating in N_2_ at 900 °C, both samples were passivated in FG at 700 °C (green square and blue circle for C-rich and O-rich respectively), to attempt to recover the *I_PL_*.

The room-temperature steady-state PL spectra for the O-rich and C-rich SiC_x_O_y_ thin films are presented in Figure 1a. The peak-to-peak PL increased ~18 times from the N_2_ 900 °C passivation to the forming gas (FG) 700 °C passivated sample in the case of the O-rich SiC_x_O_y_, whereas its C-rich counterpart exhibited a ~17 times increase for its corresponding passivated samples. Single thermal passivation in FG ambient (between 500 and 900 °C) yielded a continuous luminescence enhancement in the case of the C-rich matrix, as depicted in Figure 1b.

The largest integrated PL (*I_PL_*) increase for the C-rich SiC_x_O_y_ was ~8.4 times, as compared with the as-deposited sample, via post-deposition passivation in FG at 900 °C. For the O-rich matrix, the largest *I_PL_* increase of ~4.2 occurred at 700 °C in FG, followed by a decrease at 900 °C. This behavior may be associated with an interplay between hydrogen desorption, as assessed by Fourier transform infrared spectroscopy (FTIR) and nuclear reaction analysis (NRA) measurements, and reduced structural disorder upon passivation (e.g., passivation of dangling bonds) [18]. The passivation benefits of FG treatments are expected to be more pronounced on C-rich thin films, which exhibit higher local disorder compared to their O-rich analogs. The latter behavior is clearly reflected by the higher bond-angle disorder seen in C-rich CVD-grown SiC_x_O_y_, as observed in FTIR studies [19].

Thermal treatments in N_2_ yielded a decrease in *I_PL_* for both matrices beyond 700 °C, as shown in Figure 1c, ruling out the increase in *I_PL_* for the FG-passivated samples being due to purely thermal treatment. Instead, this suggests that the two ambients affect the observed PL differently. In an attempt to recover the observed PL after the N_2_ 900 °C passivation, a 700 °C FG-passivation was performed sequentially, shown in Figure 1c. The *I_PL_* was not recovered, suggesting that the induced structural changes via N_2_-passivation cannot be recovered with FG-passivation at this temperature.

**Figure 2:**
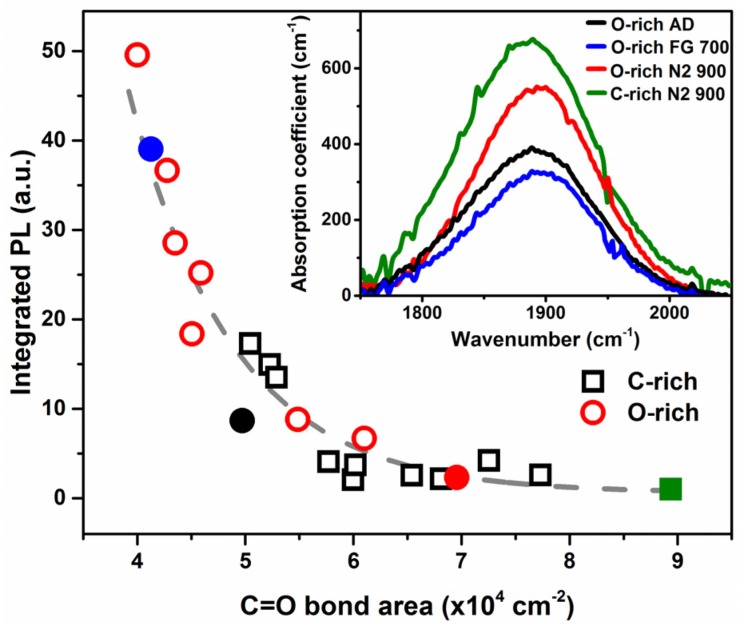
*I_PL_* of both compositions of SiC_x_O_y_ thin films and nanowires (NWs) related to the carbonyl bond area from the infrared absorption spectra. It was observed that the *I_PL_* of the films dropped in an exponential fashion with respect to the carbonyl bond area present in the material by ~50 times. Gray dashed line is a guide-to-eye. Solid blue, black, and red points correspond to the O-rich FG 700 °C, as-deposited, and N_2_ 900 °C samples respectively. Solid green square corresponds to the C-rich N_2_ 900 °C sample. Inset: Infrared absorption spectra depicting the modulation of the carbonyl bonding present in the as-deposited, FG 700 °C, and N_2_ 900 °C passivated O-rich samples, as well as the N_2_ 900 °C passivated C-rich sample.

In order to further elucidate on the behavior of the *I_PL_* from the SiC_x_O_y_ matrix with regards to the structural composition itself, a combinational and systematic FTIR and PL study was carried out. It was observed that the *I_PL_* of the films was inversely related to a bonding configuration at ~1880 cm^−1^ in the infrared spectra, as depicted in the inset of Figure 2. This bonding configuration is attributed to carbonyl (C=O) stretching mode [19], blue-shifted by nearby oxygen atoms. This PL behavior is in agreement with the observed role of C=O groups in materials, where C=O act as traps for non-radiative pathways dissociating the photo-generated carriers, and thus quenching the PL [20,21]. Therefore, the decrease in *I_PL_* with increasing C=O bond area (Figure 2) is expected, suggesting an increase in the density of non-radiative pathways related to the carbonyl bonding configuration in films, which has been also observed in graphene oxide [22].

It was found that the presence of carbonyl bonding in the films could be further modulated beyond composition control during synthesis, as thermal passivations in FG and N_2_ ambients, respectively, decreased and increased the carbonyl present in the films. The solid points representative of the modulation of the bond density of C=O in the SiC_x_O_y_ matrix correspond to the FTIR spectra of the same color depicted in the inset of Figure 2. As is highlighted in the inset of Figure 2, N_2_-passivation increased the C=O present in the O-rich matrix, whereas FG-passivation decreased the amount present with respect to its as-deposited. Through effective defect engineering by means of proper tailoring of the passivations, it was found that the density of C=O could be reduced by a factor of ~2.2, yielding a substantial non-linear PL enhancement of ~50 times (Figure 2).

**Figure 3:**
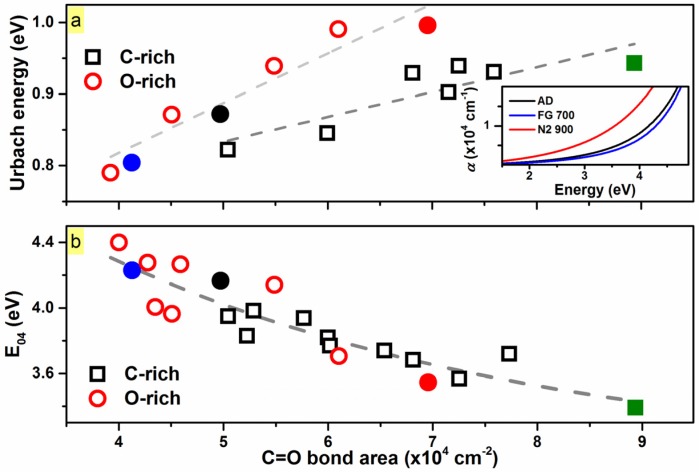
(**a**) Urbach energy (*E_u_*) of both compositions of SiC_x_O_y_ related to the carbonyl bond area from the infrared absorption spectra. *E_u_* was determined based on the following equation, $\alpha =\alpha_{0}e^{\left(\frac{E}{E_{u}}\right)}$, where *α* is the absorption coefficient, *α*_0_ is a constant, and *E_u_* is the Urbach energy. *E_u_* energies are correlated linearly and trend upward with increasing carbonyl (gray dashed lines), suggesting increasing band tail width with increasing carbonyl. Inset: The absorption coefficient (*α*) as a function of energy for the O-rich as-deposited, FG 700 °C, and N_2_ 900 °C passivated samples. Increased sub-*E_04_* gap absorption was seen in the case of the 900 °C N_2_-annealed SiC_x_O_y_ thin film; (**b**) *E_04_* of both compositions of SiC_x_O_y_ related to the carbonyl bond area. The *E_04_* values decrease with increasing carbonyl present in the films. Gray dashed line is a guide-to-eye.

Additionally, FTIR analysis revealed the presence of Si–H bonding (~2050 cm^−1^) in both as-deposited matrices [19]. Upon annealing treatments in N_2_ up to 900 °C, there was no observable change in Si–H bonding; however, passivation in FG revealed an increase in the Si–H_n_ stretching mode at ~2250 cm^−1^ [19,23], suggesting the presence of unsaturated bonds in the as-deposited and N_2_-passivated samples. Coupled with the decrease in C=O upon FG-passivation, it is therefore implied that the FG-passivation reduces the carbonyl bonding, and in parallel, saturates dangling bonds. Conversely, FTIR showed that the N_2_-passivation increases the C=O present in the matrix, and this is coupled with the absence of any Si-H_n_ bonding (2100–2250 cm^−1^), indicating that any dangling bonds in the films remained unsaturated. This would lead to enhanced structural disorder and density of defect states in films, thereby increasing the absorption (*α*). This was indeed observed by ultraviolet-visible spectroscopy ellipsometry (UV-VIS-SE) analysis (inset of Figure 3a), as increased absorption was seen in the case of the 900 °C N_2_-annealed SiC_x_O_y_.

Furthermore, sub-*E_04_* optical gap absorption involves transitions from or to localized states, and has an exponential-like dependence on the photon energy (*E_04_*: energy where the absorption coefficient, *α*, is 10^4^ cm^−1^) [24]. The tailing of the band edges is characterized by the Urbach energy, *E_u_*, and can be employed to characterize the energy width of the band-tail states in amorphous systems [25]. As shown in Figure 3a, the *E_u_* energies for the samples increases almost linearly with the increased presence of carbonyl in materials, suggesting broadening of the band-tails in SiC_x_O_y_. Accordingly, the *E_04_* gap decreased linearly with increased C=O.

Thus, the increase in *E_u_* energies, correlated to the presence of C=O in films, may be attributed to an increased disorder in SiC_x_O_y_. As discussed, the latter is suggested to be related to the formation of dangling bonds (e.g., during N_2_ annealing), which in turn is expected to result in increased absorption and lower *E_04_* values, as evidently seen in Figure 3b. This effectively reduces the probability of radiative transitions, as photo-generated carriers during thermalization would diffuse away by hopping to non-radiative recombination centers (e.g., Si dangling bonds) before they recombine [26,27]. Hence, there is an observed decrease in *I_PL_* with increasing *E_u_*.

To gain further insight into the spectral characteristics of SiC_x_O_y_ (such as possible luminescence emanating from defects/localized states (e.g., C=O defect-related states)), temperature-dependent photoluminescence (TDPL) spectra of two passivated O-rich thin films with varying C=O density were collected. This corresponds to blue and red solid points for the, respectively, FG 700 °C (low bond density of C=O) and N_2_ 900 °C (high C=O bond density) O-rich samples in Figure 2 and Figure 3.

**Figure 4:**
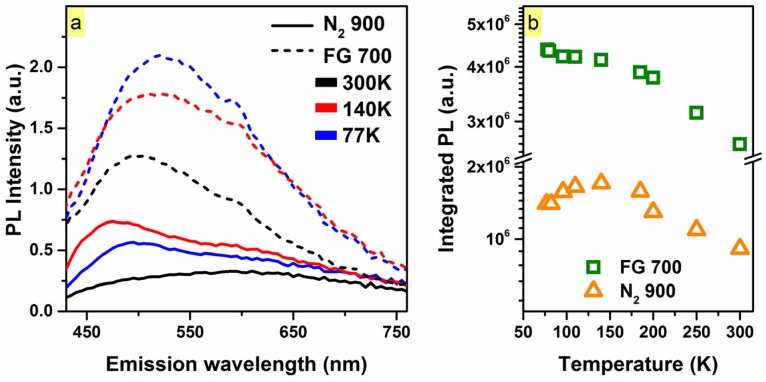
(**a**) Temperature-dependent steady-state photoluminescence spectra of the N_2_ 900 °C (solid) and FG 700 °C passivated (dashed) O-rich sample under 400 nm excitation. Two components were observed at ~470 and ~580 nm; (**b**) *I_PL_* as a function of temperature for the corresponding passivated samples. The N_2_-passivated sample exhibited stronger temperature dependence, with the *I_PL_* nearly doubling from 300 to 140 K.

Figure 4a shows the steady-state TDPL spectra for these two samples under 400 nm excitation (sub-gap excitation in the case of FG-passivated sample). Two characteristic areas of the PL spectra were observed and further probed at ~470 nm and ~580 nm. The 470 nm component exhibited stronger temperature dependence in the N_2_-passivated sample compared to its FG-passivated counterpart, exhibiting an increase in intensity of ~3 from 300 K to 140 K. The 580 nm component exhibited an increase in intensity of ~1.7 times for both of the annealed samples. As shown in Figure 4b, the total integrated PL of both samples increased ~1.5 times, from 300 K to 77 K.

The temperature-dependence behavior and red-shift (as expected with a decreasing density of states considering sub-gap excitation) of the PL of the FG-passivated sample is consistent with band-tail state luminescence [26,28]. The PL efficiency monotonically decreases as the temperature increases from 77 K to 140 K. Temperature-dependent tail-state luminescence occurs because at low temperatures (e.g., 77 K), electron-hole pairs have less chance (in contrast to high temperatures) to hop and diffuse out to adjacent energy states, making radiative recombination more likely. Consequently, the temperature dependence is more pronounced for lower band-tail states (~520 nm). These states are closer to the hopping edge and are thus less localized compared to the deepest and more localized tail states (longer wavelength).

In the case of the N_2_ 900 °C sample, *I_PL_* increased with increasing temperature from ~77 to 140 K, and then monotonically decreased until room-temperature. This negative thermal PL quenching behavior from ~77 to ~140 K [29] (i.e., the increase in *I_PL_* with an increase in temperature) is not consistent with tail-state luminescence, indicating the presence of additional defect-related localized states ~470 nm. To this end, carbonyl (C=O) groups exhibit light emission around ~470 nm, having a long PL lifetime in the ms range [30,31].

**Figure 5:**
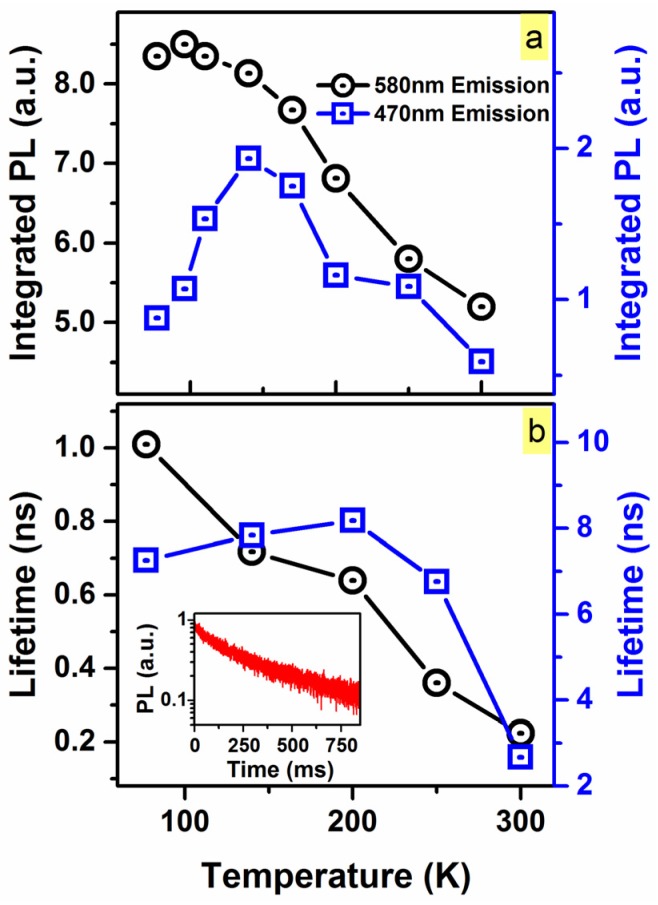
Temperature dependence behavior of (**a**) the *I_PL_* and (**b**) luminescence lifetime for the ~470 and ~580 nm components deconvoluted from the spectra of the N_2_ 900 °C passivated SiC_0.3_O_1.6_ sample. A monotonic increase in the *I_PL_* of the 580 nm component was observed as temperature was decreased, which was also reflected in the luminescence lifetime. The *I_PL_* of the 470 nm component increased until 140 K, and then decreased as temperature was lowered to 77 K. Below room temperature, the apparent luminescence lifetime of the 470 nm component plateaued around 8 ns; however, under the acquisition parameters used during the measurement, the ~8 ns extracted is not the lifetime, but is the upper limit of the measurement, with the values exhibiting a standard deviation of ± 2.2 ns below 250 K. Inset: Time-resolved photoluminescence (TRPL) spectrum of the 470 nm component acquired at 140 K with a microsecond flashlamp (repetition rate of 1 Hz)*.*

To provide further evidence into this, TDPL and TD-TRPL were utilized to probe the two emissions at 470 and 580 nm of the N_2_ 900 °C O-rich (SiC_0.3_O_1.6_) sample. The temperature-dependent PL and lifetime of the two emissions, deconvoluted from the steady-state spectra, are depicted in Figure 5. The PL intensity and lifetime from the 580 nm component monotonically decreased as temperature increased, agreeing with the band-tail state recombination model. On the contrary, the 470 nm component exhibited negative thermal quenching from ~77 to ~140 K (Figure 5a, blue), as PL intensity initially increased in this range, and then decreased until room-temperature.

This temperature-dependent PL behavior at ~470 nm further implies the presence of a C=O defect-related emission in the SiC_x_O_y_ sample with high C=O bond-density. It is suggested that at 77 K, carriers are localized at band-tails states below 2.6 eV (~470 nm). As temperature is increased to 140 K, a higher number of carriers can attain enough thermal energy to thermalize up to higher band-tail states (~2.6 eV). Thus, this has a higher probability to hop/migrate to spatially nearby C=O-related energy states to recombine radiatively. Further increases in temperature would decrease the probability of hopping/migration to C=O-related energy states, as the probability of the carriers to thermalize to lower band-tail states and/or to hope away to defects (e.g., dangling bonds) to recombine non-radiatively is significantly increased [26,32], thus decreasing the 470 nm-PL (Figure 5a).

Additionally, if the 470 nm component were to originate from the C=O present in the N_2_ 900 °C passivated sample, this would involve a triplet-to-singlet transition and would be characterized by a long lifetime. For the 470 nm component (C=O-related PL emission), the lifetime was determined to be ~8 ns below 250 K. However, this lifetime value, which exhibited a standard deviation of ±2.2 ns, is approximately the upper limit of the TRPL measurement under the acquisition parameters utilized (repetition rate of 20 MHz), suggesting a longer lifetime. In order to accurately measure the lifetime of the C=O-related PL emission (~470 nm), a microsecond flashlamp (repetition rate of 1 Hz) was employed. The PL lifetime was determined to be ~160 ms at 140 K. This long lifetime is expected from PL emission, originating from triplet to singlet C=O-related transitions [33], in agreement with our attribution of the ~470 nm-PL emission.

**Figure 6:**
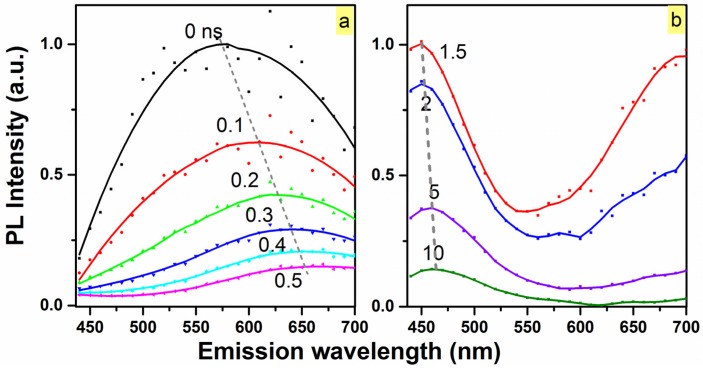
Time evolution of the steady-state PL of the N_2_ 900 °C passivated SiC_0.3_O_1.6_ sample at 77 K. (**a**) Over the first few hundreds of picoseconds, the PL of the ~580 nm component dominates the spectrum before decaying completely; (**b**) Beyond one nanosecond, the ~470 nm component persists in the spectrum up to 50 ns (measurement upper limit-20 MHz repetition rate).

Time-resolved emission spectroscopy (TRES) at 77 K was carried out to investigate the spectral evolution of the N_2_ 900 °C passivated O-rich sample (Figure 6). Within the first 500 ps, the red emission at 580 nm related to the band-tail recombination present in the matrix drops more than one order of magnitude, and it is clearly shown in Figure 6a that the emission red-shifts with time. This behavior is expected, as transitions associated with lower energy band-tail states would exhibit slower recombination rates as the thermalization rate towards lower energy states decreases significantly, due to the decreasing density of band-tail states [11]. However, beyond the first nanosecond, the emission at 470 nm persists even up to the measurement limit of 50 ns (as seen in Figure 6b), and does not appear to shift, indicative of emission from defect-related localized states.

## 3. Materials and Methods 

### 3.1. Thin Film Synthesis

SiC_x_O_y_ thin films and NWs (~180 nm thick) were synthesized in a hot-wall quartz tube furnace by TCVD at 800 °C on Si(100) substrates. Si(100) substrates were cleaned using acetone, isopropyl alcohol, deionized water, and were dried using nitrogen. System pressure was maintained between 1 to 2 Torr via a throttle valve, and the oxygen content of the films was modulated by changing the flow rate (hence partial pressure) of ultra-high purity oxygen during deposition. A non-halogenated organosilcate (2,4,6-trimethyl-2,4,6-trisilaheptane) was used as the silicon and carbon source, flown into the chamber with a vapor source mass flow controller at 10 sccm. Further experimental details for the thin film synthesis, NW fabrication, and their associated structural and optical properties can be found in prior publications by the current investigators [10,11,19].

### 3.2. Thermal Passivation of the SiC_x_O_y_ Thin Films

The carbon-rich (C-rich) SiC_0.5_O_1.3_ and oxygen-rich (O-rich) SiC_0.3_O_1.6_ amorphous thin films underwent passivation for 1 h at temperatures of 500–900 °C in ultra-high purity O_2_, N_2_ and FG. System chamber was evacuated and underwent three purge cycles using the gas for the passivation prior to starting the heating process.

### 3.3. Characterization of the SiC_x_O_y_ Thin Films

The structural and optical properties of the as-deposited and passivated samples were characterized by FTIR (Nicolet iS50, Thermo Scientific, Waltham, MA, USA) and UV-VIS-SE (GESP-5, Semilab, Budapest, Hungary). The room-temperature steady-state PL measurements were performed in an FLSP920 spectrometer from Edinburgh Instruments using a Xe lamp at 300 and 400 nm excitation (Edinburgh Instruments, Livingston, UK). Additionally, TDPL and TD-TRPL studies were conducted by utilizing a closed-loop cold finger cryostat (ST-100, Janis Research, Woburn, MA, USA). TRPL spectra were collected in the same spectrometer utilizing a time correlated single photon counting method, with a pulsed diode laser source [λ = 405 nm (3.06 eV), ~50 ps full width at half maximum (FWHM) and 20 MHz repetition rate].

## 4. Conclusions

The PL from both the as-deposited and passivated CVD-synthesized SiC_x_O_y_ materials was strong enough to be seen by the naked eye, while the PL yield was found to be inversely related to the carbonyl (C=O) bond density present in the materials. Through post-deposition passivations, the peak-to-peak PL was enhanced ~18 and ~17 times, respectively, for the O-rich and C-rich matrices in this study.

Furthermore, through optimized post-deposition FG-passivation treatments, the local bonding environment of the SiC_x_O_y_ can be modified to decrease the amount of C=O. Based on FTIR analysis, this is coupled to the effective passivation of dangling bonds in the materials. In turn, the *E_u_* and *E_04_* energies are, respectively, decreased and increased with decreasing C=O in the materials, yielding the observed substantial non-linear PL enhancement of ~50 times.
